# O-01 - EVALUATING COMPLETENESS OF SHIGA TOXIN-PRODUCING ESCHERICHIA COLI-ASSOCIATED HAEMOLYTIC URAEMIC SYNDROME SURVEILLANCE IN SCOTLAND

**DOI:** 10.1093/pubmed/fdag046.001

**Published:** 2026-07-09

**Authors:** Ms Navneet Rai, Annemarie Van Heelsum, Susan Brownlie, Genna Leckenby, Lynda Browning, Janine Thoulass, Sema Nickbakhsh

**Affiliations:** Public Health Scotland; Public Health Scotland; Public Health Scotland; Public Health Scotland; Public Health Scotland; Public Health Scotland; Public Health Scotland

## Abstract

**Background:**

In 2023, Scotland experienced an increase in haemolytic uraemic syndrome (HUS) associated with shiga toxin-producing Escherichia coli (STEC), alongside a decade-long rise in reported STEC infections. Around 10% of STEC cases develop HUS, a serious condition primarily affecting children and adults over 65. Although STEC is the predominant cause of HUS, surveillance limitations hinder timely and reliable assessment of changes in HUS trends.

**Objectives:**

We examined the completeness of HUS surveillance data available through STEC Enhanced Surveillance Questionnaires (ESQs) by comparison with national digitalised hospital admissions data, with and without laboratory data linkage, over an extended timeframe.

**Methods:**

A capture-recapture analysis compared HUS cases identified through ESQs with ICD-10 coded hospital admissions (Scottish Morbidity Records; SMR01) between 2019-2024. Hospital admissions were evaluated using two methods: (1) with linkage to laboratory records (Electronic Communication of Surveillance Scotland; ECOSS) to identify STEC-associated HUS, and (2) without ECOSS linkage to include non-STEC causes of HUS.

**Results:**

Overall 28 HUS cases were identified from ESQs, 88 from SMR01 (with ECOSS linkage), 160 from SMR01 (without ECOSS linkage), providing 156 unique HUS cases. This gave an ESQ system sensitivity, for STEC-associated HUS, of 47% among 60 unique STEC-HUS cases (60 from SMR01-ECOSS linkage). Under-ascertainment through ESQs is expected, as HUS often develops after public health follow-up. Hospital admissions linked to ECOSS records also underestimate because not all STEC infections are laboratory confirmed. In contrast, hospital admissions without laboratory records overestimate by including non-STEC causes of HUS.

**Conclusions:**

Ascertainment of STEC-associated HUS cases in Scotland is incomplete using national enhanced surveillance for STEC alone. Digitalised hospital admissions data provide more comprehensive and consistent intelligence that may strengthen situational awareness and epidemiological understanding of HUS trends. However, accurately determining the true number of HUS cases caused by STEC infection remains a challenge.

